# Supplementary material for: Content-rich biological network constructed by mining PubMed abstracts
Source: BMC Bioinformatics. 2004 Oct 8;5:147. doi: 10.1186/1471-2105-5-147 (PMC528731; doi:10.1186/1471-2105-5-147)
Supplement: Additional File 5 — The original Chilibot query results of the term "long-term potentiation (LTP)" and 22 other terms, limiting the latest references analyzed to the years 1990, 1995, 2000, and 2004. [file 1471-2105-5-147-S5.bz2 › chilibotAdditionalFile5/ltp2000/html/TRKA_AMPA.html]

 


 **TRKA** and **AMPA** 
  
Found 4 abstracts in PubMed,  **4 abstracts were retrieved and analyzed**.  


---

 Search Google  |
 PDF files only 
|  EDU domain only 

---

**Interactive relationship** (e.g. stimulation, inhibition, etc)

- Furthermore, KA stimulation resulted in an increase of  **TrkA**  expression and phosphorylation, which was blocked not only by the  **AMPA**  KA receptor antagonist 6 cyano 7 nitroquinoxaline dione and AG 879, but also by the phospholipase C inhibitor U73122 and the intracellular calcium chelator BAPTA.  Ref: 10820201 J Neurochem, 2000
- These results indicate that in developing cortical neurons, activation of  **AMPA**  KA receptors by KA may induce expression, followed by activation of  **TrkA**  via PLC signaling and intracellular calcium elevation and hence increase reception of NGF on KA challenged neurons.  Ref: 10820201 J Neurochem, 2000

- :-)

  **Parallel relationship** (e.g. studied together, co-existance, homology, etc.)

  - A study of polyphosphoinositide turnover showed that KA stimulated phospholipase C PLC activity was directly triggered by the  **AMPA**  KA receptor activity, but not by the activity of  **TrkA**  or other excitatory amino acid receptor subtypes.  Ref: 10820201 J Neurochem, 2000
  - Prior reports demonstrated that cells of the oligodendroglial lineage are susceptible to excitotoxic necrosis mediated by alpha amino 3 hydroxy 5 methylisoxazole 4 propionic acid glutamate receptors  **AMPA**  GluR, and also showed that these cells express the high affinity neurotrophin receptors, TrkC and  **TrkA** .  Ref: 10861784 J Neurosci Res, 2000
